# A novel biomarker Ins60/ApoA for predicting diabetic kidney disease in newly diagnosed type 2 diabetes: a pilot study

**DOI:** 10.3389/fmed.2025.1569730

**Published:** 2025-10-09

**Authors:** Jin Sun, Mengqing Ma, Hao Zhang, Hao Hu, Yuanyuan Liu, Wanwan Zhang, Binbin Pan, Xin Wan

**Affiliations:** ^1^Department of Endocrinology, Xuzhou First People’s Hospital, The Affiliated Xuzhou Municipal Hospital of Xuzhou Medical University, Xuzhou, China; ^2^Department of Nephrology, Nanjing First Hospital, Nanjing Medical University, Nanjing, China

**Keywords:** Ins60/ApoA, ratio of albuminuria and creatinine, newly diagnosed type 2 diabetes, diabetic kidney disease, body mass index

## Abstract

**Objective:**

We performed this cross-sectional study to explore potential biomarkers for predicting diabetic kidney disease (DKD) in newly diagnosed type 2 diabetes mellitus (T2DM).

**Methods:**

A total of 623 patients were recruited from Xuzhou First People’s Hospital and Nanjing First Hospital based on the electronic case records system. Patients were grouped according to their albuminuria-to-creatinine ratio (ACR) into two categories: ACR < 30 mg/g and ACR ≥ 30 mg/g. Biomarker levels between the two ACR groups were compared using an independent sample *t*-test. Correlation analysis was determined using Pearson’s or Spearman’s analysis and binary logistic regression. Receiver operating characteristic (ROC) curve analysis was used to elucidate the predictive effect of biomarkers on DKD.

**Results:**

The levels of total cholesterol, glycated hemoglobin (HbA1c), fasting C-peptide, fasting insulin, and prevalence of hypertension were higher, while the levels of red blood cell counts (RBC), hemoglobin, ApoA, and free triiodothyronine were lower in the ACR ≥ 30 mg/g group. Negative correlations were found between ACR ≥ 30 mg/g and RBC, hemoglobin, albumin, and NAFLD (*r* = −0.094, *p* = 0.02; *r* = −0.130, *p* = 0.001; *r* = −0.137, *p* = 0.001; *r* = −0.097, *p* = 0.018), while positive correlations were found between high-density lipoprotein, fasting blood glucose, hypertension, and the ratio of 60-min postprandial insulin and serum apolipoprotein(a) (Ins60/ApoA) (*r* = 0.134, *p* = 0.001; *r* = 0.120, *p* = 0.003; *r* = 0.131, *p* = 0.001; *r* = 0.359, *p* = 0.001). Furthermore, binary logistic regression showed that lnIns60/ApoA was an independent influence factor for ACR ≥ 30 mg/g. After adjusting for age, gender, hypertension, non-alcoholic fatty liver disease (NAFLD), hemoglobin, albumin, smoking history, alcohol consumption history, and body mass index (BMI), lnIns60/ApoA was an independent influence factor for ACR ≥ 30 mg/g(OR = 2.778, *p* = 0.015). The area under the ROC curve was 0.741 (95% CI: 0.629–0.854, *p* = 0.001) for ACR ≥ 30 mg/g. The analysis of ROC curves revealed that an optimal cutoff for ACR was 22.42 mg/g, with a sensitivity of 67.6% and a specificity of 72.1%.

**Conclusion:**

The ratio of Ins60/ApoA could be used as an alternative biomarker for predicting DKD in newly diagnosed T2DM patients.

## Introduction

Diabetes mellitus (DM) is a global burden disease, with a prevalence of over 11% in China (1). Although treatments for diabetic complications have advanced, a significant residual risk remains (2). Therefore, early diagnosis and intervention are essential for managing diabetic complications.

Diabetic kidney disease (DKD) is a prominent complication of diabetes mellitus. It has become the main pathogenesis of chronic kidney disease (CKD) (3). Clinically, DKD patients could develop end-stage renal disease more quickly, which increases payment of national medical insurance and household expenditure. It is of importance to control proteinuria to slow the progression of DKD. Nevertheless, conventional treatment could not reduce albuminuria to the normal range when DKD is diagnosed in an advanced stage, which results in an elevated residual risk of end-stage renal disease. Therefore, early diagnosis of DKD is essential for the management of diabetes.

The albumin-to-creatinine (ACR) ratio is commonly used as a biomarker to diagnose DKD and to assess treatment effects. However, elevations in ACR often occur too late for the early recognition of kidney injury. Previous studies have suggested that beta trace protein, beta-2 microglobulin, col4a3, and klotho could be used as early biomarkers of glomerular injury, while neutrophil gelatinase-associated lipocalin, kidney injury molecule-1, N-acetyl-beta-D-glucosaminidase, liver-type fatty acid-binding protein, and slc5a2, slc34a1, slc12a3, and slc4a1 could be used to assess the injury of tubule (4–6). On top of that, a total of 32 differentially methylated CpGs were observed in diabetic kidney disease in type 1 diabetes, of which 18 are located within genes differentially expressed in kidneys or correlated with pathological traits in diabetic kidney disease (7). Moreover, m6A regulators modeled with YTHDC1, METTL3, and ALKBH5 could be used to better identify early DKD (8). Immunosenescence traits (CD28null and an inverted CD4 + CD8 + ratio) may serve as early indicators of DKD progression (9). Microbiomics and transcriptomics might also supply potential biomarkers, such as *Lactobacillus johnsonii* abundance and urinary exosomes, to assess the progression of CKD (10, 11). DKD development could occur before the awareness of the determination of ACR in diabetic patients. A previous study has found that albuminuria was observed in impaired glucose tolerance patients (12). Hence, it is essential to explore probable biomarkers like fibronectin, laminin and so on to predict DKD, especially in early diabetic stage (13). It has also been proven that CKD273, including 273 urinary peptides, was used for early diagnosis of CKD and CKD risk stratification even in patients without CKD (14). However, there is still a lack of enough biomarkers for early recognition of CKD. Our study identifies a novel biomarker, Ins60/ApoA, which may serve as an early indicator of DKD progression.

## Materials and methods

### Study population

A total of 623 diabetic patients who never reported elevation of blood glucose or received any diabetic treatments were consecutively recruited from January 2018 to August 2024 in Xuzhou First People’s Hospital and Nanjing First Hospital. The inclusion criteria were: newly diagnosed T2DM and age between 18 and 80 years. The exclusion criteria were: a history of diabetes, use of any diabetic medicine or angiotensin-converting enzyme inhibitors, younger than 18 years old, heart failure, fever, liver dysfunction, pregnancy, non-ketotic hyperosmolar coma, diabetic lactic acidosis, diabetic ketoacidosis, and acute kidney injury. Clinical information was collected, including gender, history of smoking and alcohol taking, body mass index (BMI), systolic blood pressure (SBP), diastolic blood pressure (DBP), history of stroke, hypertension, and coronary artery disease (CAD).

### Laboratory measurement

We collected clinical indices via electronic medical record. Cystatin C, serum creatinine (sCr), β2microglobin, serum albumin, calcium, phosphorus, serum lipid, and proteinuria were determined using the Beckman AU5800 automatic biochemical analyzer. The level of albuminuria was determined using immunoturbidimetry, and the level of urine creatinine was determined using the enzymatic method. ACR was calculated by the ratio of albuminuria and creatinine. Glycated hemoglobin (HbA1c) was determined using high-performance liquid chromatography (BIO-RAD). Thyroid function and levels of c-peptide and insulin were tested using electrochemiluminescence assay (Siemens, Centaur XP). Blood test was performed using the Sysmex XT-1800i Automated Hematology System (Shanghai, China).

Data calculations

1. BMI = weight/(height*height).
2. Estimated glomerular filtration rate (eGFR) was calculated using the Chronic Kidney Disease Epidemiology Collaboration (CKD-EPI) 4-level race equation (15, 16). The expressions of the eGFR equation were as follows:

$$\begin{array}{l} \text{eGFR}=\mathrm{EXP}\;(\begin{array}{l} \mathrm{LN}[51]–0.328\times \mathrm{LN}[\mathrm{Scr}/88.4/0.7]\\ +\mathrm{age}\times \mathrm{LN}[0.993] \end{array})\;\\ (\text{if female and creatinine}<0.7); \end{array}$$

$$\begin{array}{l} \text{eGFR}=\mathrm{EXP}\;(\begin{array}{l} \mathrm{LN}[151]–1.210\times \mathrm{LN}[\mathrm{Scr}/88.4/0.7]\\ +\mathrm{age}\times \mathrm{LN}[0.993] \end{array})\;\\ \left(\text{if female and creatinine}\geq 0.7\right) \end{array}$$

$$\begin{array}{l} \text{eGFR}=\mathrm{EXP}\;(\begin{array}{l} \mathrm{LN}[149]–0.412\times \mathrm{LN}[\mathrm{Scr}/88.4/0.9]\\ +\mathrm{age}\times \mathrm{LN}[0.993] \end{array})\;\\ \left(\text{if male and creatinine}<0.9\right) \end{array}$$

$$\begin{array}{l} \text{eGFR}=\mathrm{EXP}\;(\begin{array}{l} \mathrm{LN}[149]–1.210\times \mathrm{LN}[\mathrm{Scr}/88.4/0.9]\\ +\mathrm{age}\times \mathrm{LN}[0.993] \end{array})\;\\ (\text{if male and creatinine}\geq 0.9). \end{array}$$

### Definition of non-alcoholic fatty liver disease (NAFLD)

NAFLD was diagnosed based on the presence of a characteristic ultrasound (US) pattern, including diffuse hyperechogenicity of the liver parenchyma compared to the kidney cortex, deep beam attenuation, and poor visualization of the intrahepatic vessels and the diaphragm border (17). Liver ultrasonography was performed after overnight fasting by an experienced sonographer blinded to patients’ clinical and laboratory data using a 3.5- to 5-MHz convex probe. The study image was then analyzed by another sonographer who was also blinded to the patients’ clinical and laboratory data, and the final assignment to a US category (NAFLD yes/no) was performed by agreement of the two operators.

### ACR group definition

The ACR group was divided into the ACR < 30 mg/g group and the ACR ≥ 30 mg/g group.

### Definition of newly diagnosed T2DM with albuminuria (ND-DKD)

Newly diagnosed T2DM patients were diagnosed according to the OGTT test or two times of elevations of fasting blood glucose ≥7 mmoL/L, postprandial 2-h blood glucose ≥11.1 mmoL/L, or random blood glucose ≥11.1 mmoL/L without a diabetic history.

Patients with newly diagnosed T2DM and ACR ≥ 30 mg/g were defined as ND-DKD.

### Statistical analysis

Statistical analysis was performed using PASW 22.0 statistical software (SPSS Inc., Chicago, IL, USA). Data are expressed as mean ± *SD*. An independent-samples t-test was used to compare the means of ACR groups. Pearson’s or Spearman’s correlation analysis was used to determine correlations between indices. In addition, binary logistic regression was used to explore independent influence factors for ND-DKD. A receiver operating characteristic (ROC) curve was built to evaluate the predictive ability of biomarkers on ND-DKD. The optimal cutoff for the ROC curve was calculated using the Youden index. A *p*-value of < 0.05 was considered to be statistically significant.

## Results

### Characteristics of the ACR < 30 mg/g and ACR ≥ 30 mg/g groups

A total of 623 newly diagnosed type 2 diabetes mellitus patients were analyzed. There was no significant difference between the two groups in age, BMI, SBP, DBP, counts of white blood cells and platelets, levels of eGFR, apolipoprotein (B), low-density lipoprotein, triglyceride, and serum creatinine. No significant differences were observed between the two groups in concentrations of C-peptide at 30 min, 60 min, and 120 min, as well as insulin at 30 min, 60 min, and 120 min. Lower levels of red blood cell counts (RBC), hemoglobin, ApoA, and free triiodothyronine were observed in the ACR ≥ 30 mg/g group. Higher levels of high-density lipoprotein, total cholesterol, HbA1c, fasting C-peptide, fasting insulin, and Ins60/ApoA were observed in the ACR ≥ 30 mg/g group than in the ACR < 30 mg/g group (Table 1).

**Table 1:** Characteristics of ACR <30 mg/g and ACR ≥ 30 mg/g groups.

Index	ACR < 30 mg/g	30 mg/g ≤ ACR	*t*	*p*
Age	50.3 ± 13.4	51.7 ± 14.3	−1.236	0.217
BMI	25.7 ± 3.5	25.7 ± 4.5	−0.086	0.932
SBP	134.1 ± 16.6	133.2 ± 16.0	−0.031	0.975
DBP	83.6 ± 10.4	84.7 ± 10.4	−1.196	0.232
WBC	6.5 ± 1.9	6.5 ± 1.7	0.202	0.84
RBC	4.8 ± 0.5	4.7 ± 0.6	2.291	0.022*
Hemoglobin	145.6 ± 15.1	141.4 ± 17.8	3.017	0.003#
Platelet	217.7 ± 55.8	216.6 ± 61.9	0.235	0.814
eGFR	117.0 ± 17.0	118.2 ± 22.7	−0.731	0.465
ApoB	1.05 ± 0.27	1.06 ± 0.30	−0.264	0.792
ApoA	1.53 ± 0.36	1.38 ± 0.43	4.611	<0.01#
LDL	3.14 ± 0.85	3.19 ± 0.99	−0.712	0.477
HDL	1.13 ± 0.34	1.20 ± 0.42	−2.365	0.018*
Tg	2.25 ± 2.64	2.64 ± 3.35	−1.579	0.115
TC	5.02 ± 1.29	5.26 ± 1.67	−2.038	0.042*
Scr	57.3 ± 13.1	55.4 ± 18.6	1.378	0.169
HbA1c	9.7 ± 2.7	10.3 ± 2.9	−2.509	0.012*
FCP	2.44 ± 1.30	2.73 ± 1.80	−2.049	0.041*
CP-30	3.54 ± 7.63	3.48 ± 2.60	0.105	0.917
Cp-60	4.23 ± 2.95	4.53 ± 3.69	−1.01	0.313
Cp-120	5.78 ± 3.61	6.01 ± 4.76	−0.608	0.543
FINS	9.72 ± 7.04	15.9 ± 14.3	−2.66	0.01*
INS-30	26.4 ± 30.8	39.9 ± 50.7	−1.486	0.141
INS-60	37.9 ± 48.0	59.1 ± 66.5	−1.719	0.089
INS-120	5.8 ± 3.6	6.0 ± 4.8	−1.439	0.153
TSH	2.97 ± 5.74	2.45 ± 1.59	1.301	0.194
FT3	4.62 ± 0.90	4.40 ± 0.86	3.314	0.001*
FT4	17.1 ± 3.09	17.56 ± 2.36	−1.842	0.066
INS60/ApoA	20.21 ± 14.41	47.81 ± 46.08	3.365	0.002^#^

BMI, body mass index; SBP, systolic blood pressure; DBP, diastolic blood pressure; WBC, white blood cell counts; RBC, red blood cell counts; eGFR, estimated glomerular filtration rate; LDL, low-density lipoprotein; HDL, high-density lipoprotein; Tg, triglyceride; TC, total cholesterol; Scr, serum creatinine; FCP, fasting C-peptide. ^*^p < 0.05,^#^*p* < 0.01.

The frequencies of male gender, smoking history, alcohol consumption history, CAD, and stroke were comparable between the groups. However, a lower frequency of NAFLD and a higher frequency of hypertension were observed in the ACR ≥ 30 mg/g group than in the other group (Table 2).

**Table 2:** Comparison of category variances in different ACR groups.

Category variances	Gender (male)	Smoking history	Alcohol consumption history	NAFLD	CAD	Stroke	Hypertension
ACR < 30 mg/g	267(67.8%)	103(26.1%)	85(21.6%)	221(58%)	27(6.9%)	61(15.5%)	120(30.5%)
30 mg/g ≤ ACR	151(64.3%)	59(25.2%)	46(19.7%)	104(45.4%)	16(6.9%)	37(15.9%)	97(41.5%)
χ^2^	0.814	0.066	0.297	9.018	0.00	0.014	7.85
*p*	0.367	0.797	0.586	0.003^#^	0.999	0.905	0.005^#^

NAFLD, non-alcoholic fatty liver disease; CAD, coronary artery disease.

### Correlations between elevated ACR and clinical indices

Negative correlations were observed between ACR ≥ 30 mg/g and RBC, hemoglobin, albumin, and NAFLD (*r* = −0.094, *p* = 0.02; *r* = −0.130, *p* = 0.001; *r* = −0.137, *p* = 0.001; *r* = −0.097, *p* = 0.018). Positive correlations were observed between ACR ≥ 30 mg/g and high-density lipoprotein, fasting blood glucose, hypertension, and the ratio of 60-min postprandial insulin and serum apolipoprotein(a) (Ins60/ApoA) (*r* = 0.134, *p* = 0.001; *r* = 0.120, *p* = 0.003; *r* = 0.131, *p* = 0.001; *r* = 0.359, *p* = 0.001) (Table 3).

**Table 3:** Correlation analysis of elevated ACR with other indices.

Index	RBC	Hemoglobin	albumin	HDL	FBG	NAFLD	HP	INS60/ApoA
*r*	−0.094	−0.130	−0.137	0.134	0.120	−0.097	0.131	0.359
*p*	0.02^*^	0.001^#^	0.001^#^	0.001^#^	0.003^#^	0.018^*^	0.001^#^	0.001^#^

RBC, red blood cell counts; HDL, high-density lipoprotein; FBG, fasting blood glucose; NAFLD, non-alcoholic fatty liver disease; Hp, hypertension; INS60/ApoA, ratio of insulin 60 min and ApoA. ^*^P < 0.05, ^#^*P* < 0.01.

**Figure 1:**
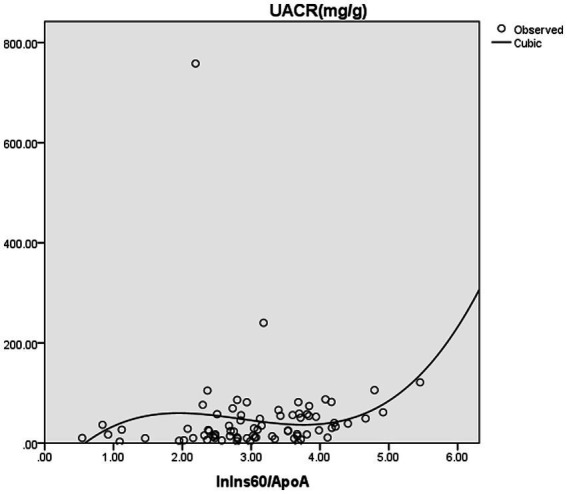
The estimated curve of ln(Ins60/ApoA) for ACR. Binary logistic regression analysis was performed with ACR ≥ 30 mg/g or not as dependent variances. The result showed that ln(Ins60/ApoA) was an independent risk factor for ACR ≥ 30 mg/g. After adjusting for age, gender, hemoglobin, albumin, NAFLD, hypertension, history of smoking, history of alcohol consumption, and BMI, ln(Ins60/ApoA) was an independent influence factor for ACR ≥ 30 mg/g (OR = 2.778, p = 0.015). The figure shows the estimated curve of ln(Ins60/ApoA) for ACR.

Furthermore, binary logistic regression analysis was performed with ACR ≥ 30 mg/g or not as the dependent variable. The result showed that ln(Ins60/ApoA) was an independent risk factor for ACR ≥ 30 mg/g. After adjusting for age, gender, hemoglobin, albumin, NAFLD, hypertension, history of smoking, history of alcohol consumption, and BMI, ln(Ins60/ApoA) was an independent influence factor for ACR ≥ 30 mg/g (OR = 2.778, *p* = 0.015) (Table 4). The estimated curve of ln(Ins60/ApoA) for ACR is shown in Figure 1.

**Table 4:** Binary logistic analysis of influence factors of ACR > 30 mg/g in newly diagnosed diabetes.

Index	OR	95%CI	*p* value
Gender	1.917	0.339–10.825	0.461
Age	0.995	0.954–1.038	0.826
Hemoglobin	0.990	0.926–1.059	0.778
Albumin	1.010	0.823–1.240	0.925
NAFLD	0.316	0.080–1.239	0.098
BMI	1.171	0.997–1.374	0.054
Hypertension	2.099	0.551–7.997	0.277
Smoking history	0.727	0.128–4.134	0.719
Alcohol consumption history	0.266	0.049–1.434	0.123
Ln(Ins60/ApoA)	2.778	1.217–6.339	0.015*

NAFLD, non-alcoholic fatty liver disease; BMI, body mass index. ^*^p < 0.05.

### The efficiency of Ins60/ApoA to predict ND-DKD

**Figure 2:**
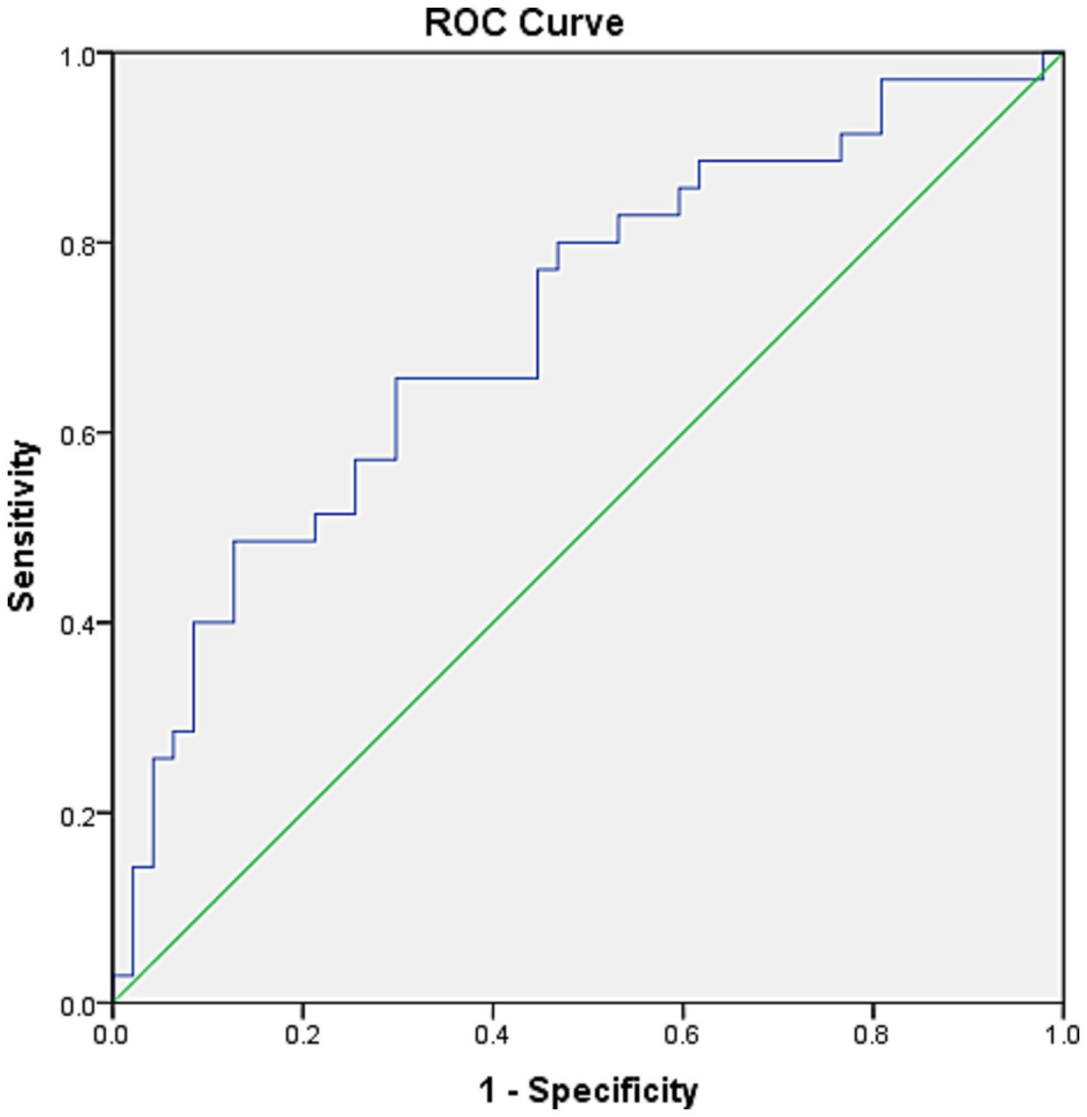
ROC curves for Ins60/ApoA for the prediction of ACR. The ROC curve was determined to evaluate the prediction efficiency of Ins60/ApoA on ND-DKD. The area under the ROC curve was 0.741 (95% CI: 0.629–0.854, *p* = 0.001) for Ins60/ApoA. The analysis of ROC curves revealed that the best cutoff for Ins60/ApoA was 22.42, with a sensitivity of 67.6% and a specificity of 72.1%.

The ROC curve was determined to evaluate the prediction efficiency of Ins60/ApoA on ND-DKD. The area under the ROC curve was 0.741 (95% CI: 0.629–0.854, *p* = 0.001) for Ins60/ApoA. The analysis of ROC curves revealed that the best cutoff for Ins60/ApoA was 22.42, with a sensitivity of 67.6% and a specificity of 72.1% (Figure 2).

## Discussion

In our study, we investigated the relationship between Ins60/ApoA and ND-DKD. The levels of Ins60/ApoA were increased, accompanied by increased levels of ACR. Furthermore, Pearson’s analysis revealed a positive correlation between Ins60/ApoA and ACR ≥ 30 mg/g. Moreover, binary logistic regression showed a significant OR value for Ins60/ApoA and BMI in influencing ND-DKD after adjusting for other clinical indices. On top of that, we performed an ROC curve to observe the predictive ability of Ins60/ApoA on ND-DKD, and the result exhibited a sensitivity of 65.7% and a specificity of 70.2%, suggesting that Ins60/ApoA was a potential biomarker for predicting ACR ≥ 30 mg/g in ND-DKD patients.

DKD is a common cause of ESRD all over the world (18). The pathogenesis of DKD is complex, including TGF-beta1, Wnt/beta-catenin signaling pathway, podocyte injury, epithelial-to-mesenchymal transition, and renin–angiotensin–aldosterone system (19–22). In recent years, dysbiosis of gut microbiota has also been reported to be associated with the development and progression of DKD (23, 24). Treating these mechanisms could slow the progression of DKD. Ophiocordyceps sinensis (OS) preparations combined with renin–angiotensin system inhibitors could decrease proteinuria in DKD patients (25). Sodium glucose cotransporter inhibitors, mineralocorticoid receptor antagonists (finerenone), and Huangkui capsules were also used to improve levels of albuminuria clinically. As *Lactobacillus* species has been identified as a method to improve the prognosis of membrane nephropathy via activation of the intrarenal AhR signaling pathway, it could be deemed as a potential treatment regimen for DKD (22). However, treatment of DKD still leaves a residual risk of ESRD or atherosclerotic cardiovascular disease (26). Early diagnosis of DKD, such as using phosphatidylcholine, lysophosphatidylcholine, and lysophosphatidic acid, could give more benefits for DM patients (27).

The diagnosis of DKD depends on ACR or a kidney biopsy. Kidney biopsy is performed to accurately diagnose kidney disease, especially when a patient is suspicious of primary glomerulonephritis. As it is a kind of invasive procedure, a kidney biopsy cannot be used as a routine tool to diagnose DKD. ACR is a convenient method to test levels of albuminuria to determine DKD diagnosis and assess treatment effect. Clinical diagnosis of DKD relies on the duration of diabetes, for example, a 10-year period for type 1 diabetes mellitus. However, the kidney injury in type 2 diabetes mellitus varies in different patients, lacking a strict time period (28). A previous study has even reported kidney injury in impaired glucose tolerance based on the pathology of kidney (12). Therefore, it is important to explore biomarkers to predict the occurrence of DKD.

Our study collected patients with newly diagnosed diabetes to explore correlation of biomarkers and DKD. To our astonishment, the frequency of DKD was as high as 234 cases, suggesting that DKD actually occurs even in the early stages of diabetes. Furthermore, metabolism biomarkers and frequency of hypertension in patients with DKD were worse than those in the ACR < 30 mg/g group, for example, taking ApoA, HbA1c, and total cholesterol. However, the frequency of NAFLD was lower in the DKD group with comparable BMI levels between the two groups. NAFLD is a metabolic disorder characterized by excessive triglyceride deposition in the liver, which is usually accompanied by insulin resistance. Our previous study also revealed that CKD-NAFLD patients had lower levels of 24-h proteinuria, suggesting that NAFLD may be a protective factor for ACR (17). More studies are essential to analyze this kind of phenomenon in CKD patients. Levels of fasting insulin and C-peptide were also higher in the DKD group, indicating more severe insulin resistance in DKD patients (29). Based on the significant differences in metabolic biomarkers between ND-DKD and newly diagnosed type 2 diabetes mellitus patients, our study selected insulin, C-peptide, and lipoproteins to explore potential biomarkers for predicting DKD (30). After trying different combinations, Ins60/ApoA was significantly elevated in the ND-DKD group compared with the other group.

Our study further analyzed the correlation between Ins60/ApoA and ND-DKD. Levels of Ins60/ApoA are elevated parallel with ACR. The binary logistic regression also showed that Ins60/ApoA was an independent risk factor of ND-DKD. Ins60/ApoA is a novel biomarker to influence DKD, which was reported for the first time. To assess whether it could be used to predict ND-DKD, our study performed ROC analysis. The result showed that the area under the ROC curve was 0.714, with a sensitivity of 65.7% and a specificity of 70.2%, suggesting that Ins60/ApoA had a pretty good predictive ability.

The traditional diagnosis of diabetes depends on levels of fasting or postprandial blood glucose, while the International Diabetes Federation has recommended 1-h post-load plasma glucose >8.6 mmoL/L as a criterion for the diagnosis of diabetes (31). Many studies have proved 1-h postprandial blood glucose has a stronger predictive effect (AUC = 0.75, sensitivity 75%, specificity 68%) on developing diabetes and a better correlation with diabetic complications than fasting blood glucose and 2-h postprandial blood glucose (32–38). Compared with plasma glucose >8.6 mmoL/L, 1-h post-load blood glucose less than 8.6 mmoL/L had higher GFR and lower risk of developing albuminuria (39–41). As elevation of blood glucose results from impaired insulin sensitivity, increased levels of insulin at 60 min could cause more severe blood glucose fluctuation, which could promote the occurrence of diabetic complications. Therefore, Ins60/ApoA is a potential biomarker to predict DKD.

### Limitations

There are some limitations in the present study. First, our study did not analyze the effect of medicine on ACR, such as renin–angiotensin–aldosterone system inhibitors. However, renin–angiotensin–aldosterone system inhibitors could decrease levels of ACR, resulting in a lower frequency of patients in the ACR ≥ 30 mg/g group. If our study included renin–angiotensin–aldosterone system inhibitors in our analysis, there could be a more significant difference between the two groups. Second, our study did not test ACR two times to get a mean level. As we had excluded patients with fever, acute kidney injury, and other factors that could induce pseudo-elevation of ACR, elevated ACR could be used as a diagnostic criterion for ND-DKD. Finally, a lack of artificial intelligence restricted the application of the biomarker. Artificial intelligence is currently applied in many disease models to help in the diagnosis and treatment of diseases (42). It can also be used to identify novel biomarkers for the early diagnosis of DKD.

Taken together, our study revealed a significant elevation of Ins60/ApoA in newly diagnosed type 2 diabetes mellitus patients, identifying it as an independent risk factor of ND-DKD. Ins60/ApoA may serve as a novel biomarker for predicting ND-DKD.

## Data Availability

The raw data supporting the conclusions of this article will be made available by the authors, without undue reservation.
