# Supplementary material for: A novel biomarker Ins60/ApoA for predicting diabetic kidney disease in newly diagnosed type 2 diabetes: a pilot study
Source: Front Med (Lausanne). 2025 Oct 9;12:1569730. doi: 10.3389/fmed.2025.1569730 (PMC12546220; doi:10.3389/fmed.2025.1569730)
Supplement: Supplementary file 1 [file Table_1.DOC]

Table S1 Binary logistic analysis of influence factors of ACR>30mg/g in newly diagnosed diabetes with Fins/ApoA

|  | OR | 95%CI | P value |
| --- | --- | --- | --- |
| Gender | 0.572 | 0.135-2.424 | 0.448 |
| Age | 1.006 | 0.966-1.047 | 0.774 |
| Hemoglobin | 0.972 | 0.922-1.024 | 0.286 |
| Albumin | 1.043 | 0.876-1.242 | 0.636 |
| NAFLD | 0.339 | 0.097-1.187 | 0.091 |
| BMI | 1.124 | 0.961-1.315 | 0.144 |
| Hypertension | 2.599 | 0.852-7.927 | 0.093 |
| Smoking history | 0.896 | 0.239-3.367 | 0.871 |
| Alcohol drink history | 0.362 | 0.097-1.355 | 0.131 |
| FIns/ApoA | 1.108 | 0.995-1.234 | 0.063 |

FIns, fast insulin; NAFLD, non-alcoholic fatty liver disease; BMI, body mass index.
