# Supplementary material for: A novel biomarker Ins60/ApoA for predicting diabetic kidney disease in newly diagnosed type 2 diabetes: a pilot study
Source: Front Med (Lausanne). 2025 Oct 9;12:1569730. doi: 10.3389/fmed.2025.1569730 (PMC12546220; doi:10.3389/fmed.2025.1569730)
Supplement: Supplementary file 2 [file Table_2.DOC]

Table S2 Binary logistic analysis of influence factors of ACR>30mg/g in newly diagnosed diabetes with Ins30/ApoA

|  | OR | 95%CI | P value |
| --- | --- | --- | --- |
| Gender | 1.755 | 0.312-9.869 | 0.523 |
| Age | 0.980 | 0.934-1.029 | 0.417 |
| Hemoglobin | 0.997 | 0.934-1.066 | 0.938 |
| Albumin | 1.012 | 0.826-1.239 | 0.908 |
| NAFLD | 0.192 | 0.041-0.900 | 0.036^*^ |
| BMI | 1.204 | 1.020-1.421 | 0.029^*^ |
| Hypertension | 2.230 | 0.539-9.227 | 0.268 |
| Smoking history | 0.714 | 0.119-4.272 | 0.712 |
| Alcohol drink history | 0.342 | 0.059-1.966 | 0.229 |
| Ins30/ApoA | 1.037 | 0.999-1.077 | 0.057 |

Ins30, insulin 30 minutes; NAFLD, non-alcoholic fatty liver disease; BMI, body mass index. ^*^*p*<0.05.
