# Supplementary material for: A novel biomarker Ins60/ApoA for predicting diabetic kidney disease in newly diagnosed type 2 diabetes: a pilot study
Source: Front Med (Lausanne). 2025 Oct 9;12:1569730. doi: 10.3389/fmed.2025.1569730 (PMC12546220; doi:10.3389/fmed.2025.1569730)
Supplement: Supplementary file 3 [file Table_3.DOC]

Table S3 Binary logistic analysis of influence factors of ACR>30mg/g in newly diagnosed diabetes with Ins60/Scr

|  | OR | 95%CI | P value |
| --- | --- | --- | --- |
| Gender | 1.340 | 0.250-7.192 | 0.733 |
| Age | 0.996 | 0.957-1.036 | 0.837 |
| Hemoglobin | 0.989 | 0.927-1.055 | 0.731 |
| Albumin | 1.023 | 0.836-1.252 | 0.825 |
| NAFLD | 0.316 | 0.085-1.173 | 0.085 |
| BMI | 1.212 | 1.045-1.405 | 0.011^*^ |
| Hypertension | 2.114 | 0.574-7.792 | 0.260 |
| Smoking history | 0.629 | 0.114-3.480 | 0.595 |
| Alcohol drink history | 0.254 | 0.047-1.357 | 0.109 |
| Ins60/Scr | 3.873 | 0.960-15.261 | 0.057 |

Ins60, insulin 60 minutes; Scr, serum creatinine; NAFLD, non-alcoholic fatty liver disease; BMI, body mass index. ^*^*p*<0.05.
